# Social Engagement and Cognitive Health: The Mediating Role of Cognitive and Physical Activity

**DOI:** 10.1093/geroni/igaa057.914

**Published:** 2020-12-16

**Authors:** Takashi Amano, Nancy Morrow-Howell, Sojung Park, Brian Carpenter

**Affiliations:** 1 Rutgers University-Newark, Newark, New Jersey, United States; 2 Washington University in St. Louis, St. Louis, Missouri, United States

## Abstract

This study aimed to assess the association between social engagement and conversion from Cognitive Impairment No Dementia (CIND) to dementia and to investigate the mediating role of cognitive and physical engagements on that relationship. Data from two waves (2010 and 2014) of the psychosocial and core modules of the Health and Retirement Study (HRS) were used. The sample consisted of 929 people who had CIND in 2010 and participated in the survey in 2014. Latent Class Analysis (LCA) with eight indicators of social engagement (activities with children, volunteering with youth/others, attending educational course/organization, meeting up, speaking on the phone, writing or emailing) found three groups: formal and informal social engagement (20.7%), informal social engagement only (48.9%), and low social engagement (30.5%). Binary logistic regression analysis showed sub groups with higher levels and greater variety of social engagements were associated with lower probability of conversion to dementia in four years. Path analysis with structural equation modeling (SEM) framework showed the relationship between patterns of social engagement and lower conversion to dementia was mediated by having higher engagement in cognitive activities (e.g. home maintenance, playing sports), but not by engagement in physical activities (e.g. playing games, using computer). Results from this study implied (1) promoting active social engagement may be a promising intervention to prevent or delay conversion from CIND to dementia, and (2) promoting social engagement may be a particularly effective and efficient strategy since it promotes other activity engagements that may itself prevent or delay conversion from CIND to dementia.

